# The prognostic role of preoperative serum albumin/globulin ratio in patients with non-metastatic renal cell carcinoma undergoing partial or radical nephrectomy

**DOI:** 10.1038/s41598-020-68975-3

**Published:** 2020-07-20

**Authors:** Jae-Wook Chung, Dong Jin Park, So Young Chun, Seock Hwan Choi, Jun Nyung Lee, Bum Soo Kim, Hyun Tae Kim, Tae-Hwan Kim, Eun Sang Yoo, Seok-Soo Byun, Eu Chang Hwang, Seok Ho Kang, Sung-Hoo Hong, Jinsoo Chung, Cheol Kwak, Yong- June Kim, Yun-Sok Ha, Tae Gyun Kwon

**Affiliations:** 1 Department of Urology, Kyungpook National University Chilgok Hospital, School of Medicine, Kyungpook National University, 807 Hoguk-ro, Buk-gu, Daegu, Republic of Korea; 2 Department of Urology, Seoul National University College of Medicine, Seoul National University Bundang Hospital, Seongnam, South Korea; 3 Department of Urology, Chonnam National University Medical School, Gwangju, Jeonnam South Korea; 4 Department of Urology, Korea University School of Medicine, Seoul, South Korea; 5 Department of Urology, College of Medicine, The Catholic University of Korea, Seoul, South Korea; 6 Department of Urology, National Cancer Center, Goyang, South Korea; 7 Department of Urology, Seoul National University College of Medicine, Seoul, South Korea; 8 Department of Urology, Chungbuk National University College of Medicine, Cheongju, South Korea

**Keywords:** Cancer, Oncology, Urology

## Abstract

This multi-institutional study sought to clarify the association between the preoperative serum albumin/globulin ratio (AGR) and the prognosis of renal cell carcinoma (RCC) in a large cohort. This study encompassed eight institutions and 2,970 non-metastatic RCC patients who underwent a radical or partial nephrectomy from the Korean RCC (KORCC) database. A low AGR (1,143 patients; 38.5%) was defined as a preoperative AGR of less than 1.47 and a high AGR (1,827 patients; 61.5%) was defined as that 1.47 or greater. In the low AGR group, older age, female gender, the incidence of symptom presentation when diagnosed, diabetes, and hypertension was higher than in the high AGR group. Patients with low AGRs showed more progressive tumor stages with higher Fuhrman nuclear grades (all P-values < 0.05). Patients in the low AGR group had a significantly lower overall survival rate (OS) and recurrence-free survival rate (RFS) in the Kaplan–Meier curves (all P-values < 0.05). AGR was an independent prognostic factor for predicting the OS and RFS in the multivariate analysis (all P-values < 0.05). The preoperative AGR is approachable and economical to use clinically for estimating the prognosis of RCC patients treated with surgery.

## Introduction

Renal cancer is the second most frequent urogenital tumor^1,2^ after bladder cancer and is occupying approximately 2.2% of all malignant tumors worldwide, with 403,262 new cases diagnosed in 2018 and around 175,098 deaths per year^3^. Of all renal cancers, renal cell carcinoma (RCC) makes up nearly 80–85%^4^. In 2019, Cases for newly diagnosed renal cancer are estimated to 73,820 in the United States^5^. And renal cancer is the 6th most frequently detected primary cancer in males, and 8th in females^5^. In Korea, 5-year survival rate for kidney cancer patients has increased over the last few decades^6^.

The most frequently detected pathologic subtype of RCC is clear cell RCC and it makes up 70–80% of all newly diagnosed RCC. It is well known that RCC is not sensitive to chemotherapy or radiation therapy. An effective treatment for clinically localized RCC is surgical resection; however, after surgical resection, approximately 20–30% of localized cancer patients face relapse^7^. Many researchers have conducted several studies to determine factors to accurately predict recurrence and prognosis in patients with RCC^8^. Among the prognostic factors, clinical prognostic factors have less evidence compared with anatomical or histological prognostic factors^9^. However, preoperative laboratory measurements from blood, especially, are inexpensive and easily measured using standardized approaches^8,10^. To date, studies on various laboratory measurements from the blood have been published^10^.

Albumin comprises 55% of serum protein. It is associated with nutritional state and is a marker for systemic inflammation in cancer patients^11,12^. Globulin, which molecular weight is higher than albumin, is the protein binding to cortisol and plays an essential part in immunity as well as inflammation^13,14^. Hypoalbuminemia, as well as hyperglobulinemia, could work in markers of chronic inflammation^15,16^. Since the albumin to globulin ratio (AGR), which is computed as albumin divided by the value of total protein minus albumin, combines the inflammatory as well as the nutritional factor in one measure, it is a good indicator reflecting these two factors^17^. A low preoperative AGR is related to worse prognosis in various human cancers in a meta-analysis^18^.

To date, three single-center-based studies have evaluated preoperative AGR as a predictive factor in RCC patients^17,19,20^. In a large multicenter setting, however, few kinds of research evaluated the predictive effect of preoperative AGR in RCC. Thus, the current study attempts to determine whether preoperative AGR is related to the prognosis for non-metastatic RCC in a large multicenter setting in Korea.

## Results

**Figure 1:**
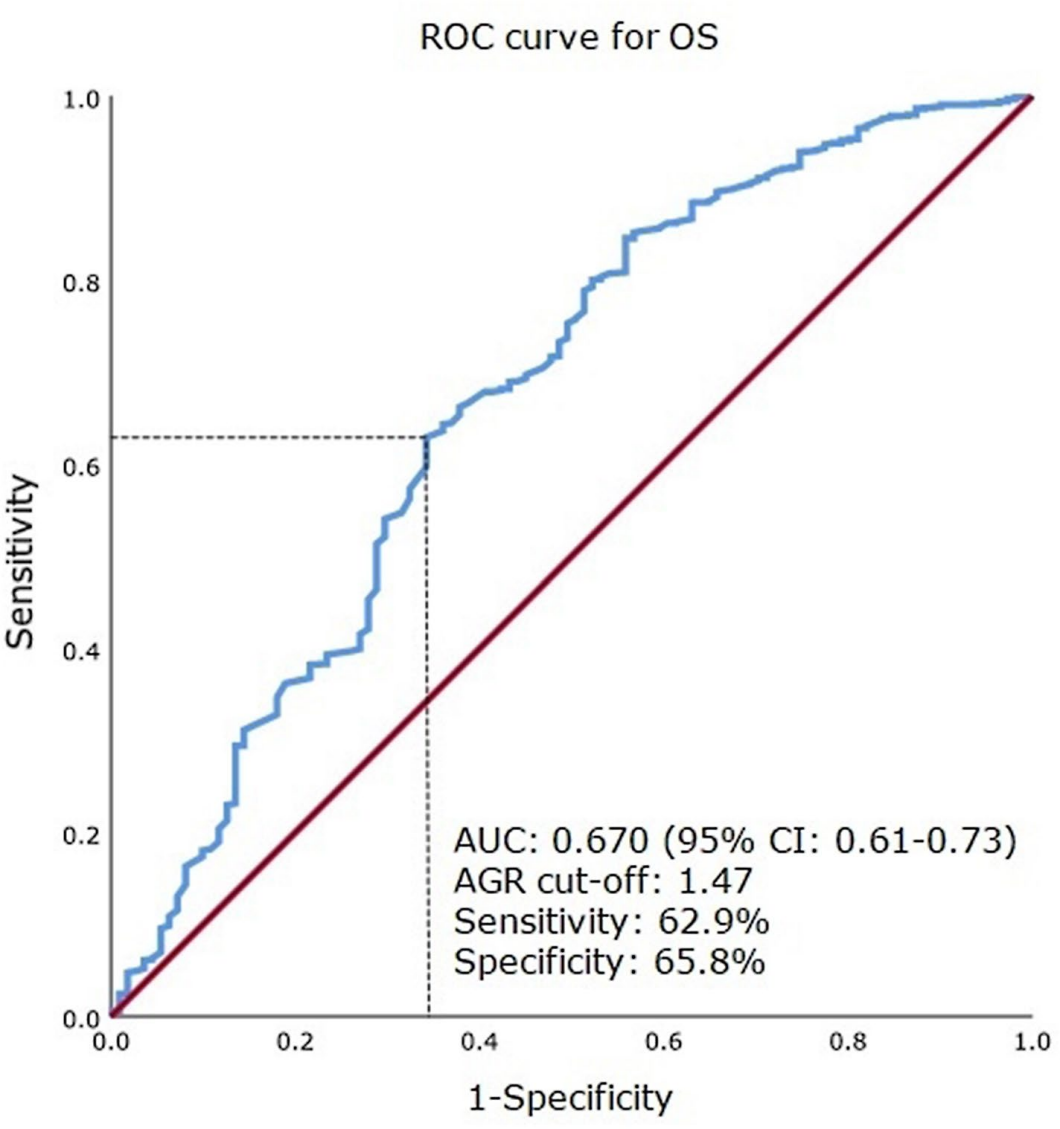
Best cut-off value for AGR was applied with ROC curves for OS. AGR, albumin–globulin ratio; ROC, receiver operating characteristic; OS, overall survival.

**Figure 2:**
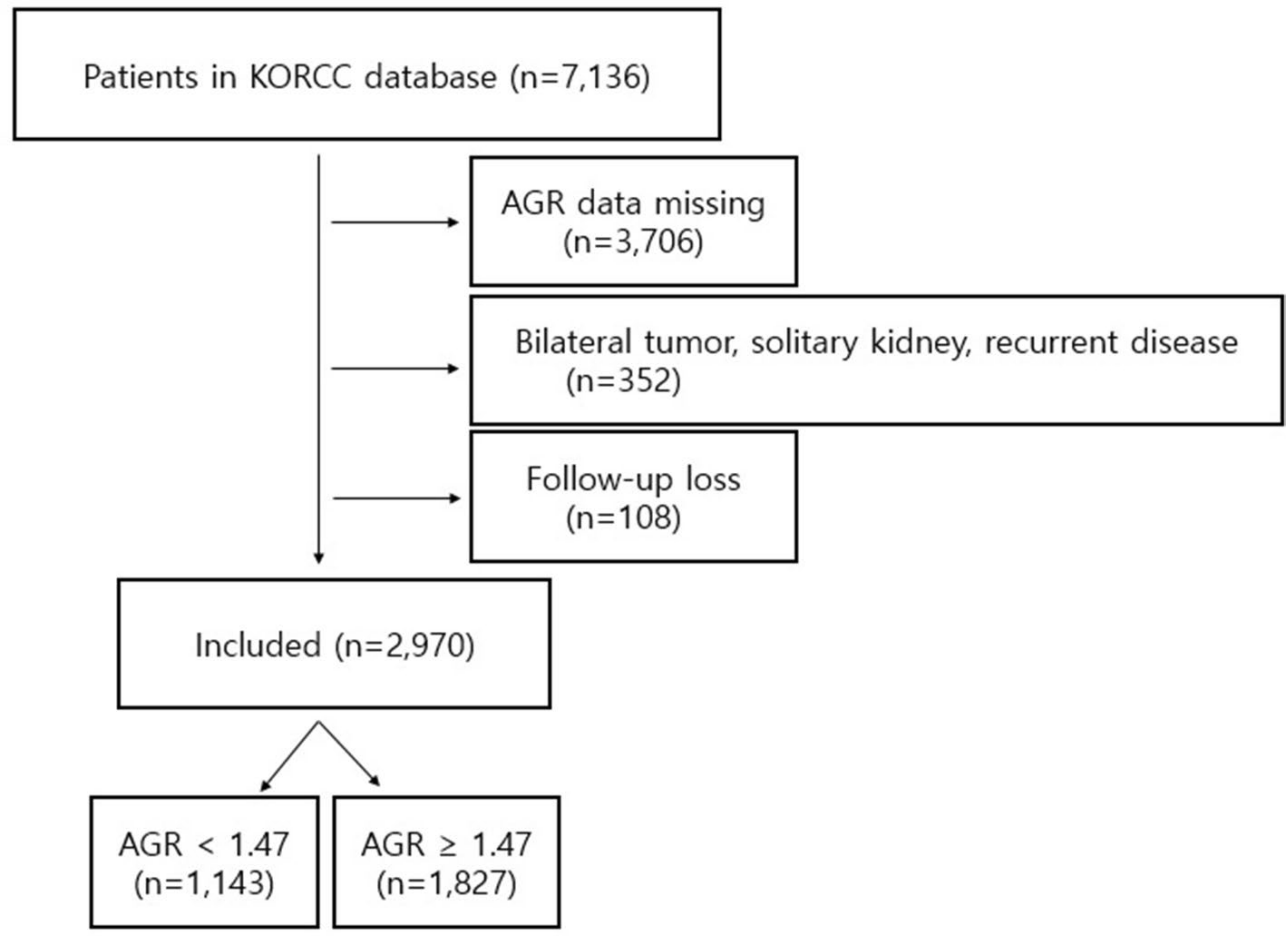
Flowchart diagram. Of the 7,136 patients in the KORCC database, 3,706 patients were excluded due to missing preoperative AGR data and 460 patients did not meet the inclusion criteria listed on the right side of the diagram. Finally, a total of 2,970 patients were included for the analysis, of whom 1,143 patients had an AGR of less than 3 and 1,827 had an AGR of 3 or greater.

The best cut-off value of AGR for overall survival (OS) was computed to be 1.47 in accordance with the receiver operating characteristic (ROC) curve. And the area under the ROC curve was 0.670 (95% CI 0.613–0.727; P < 0.001) (Fig. 1). Figure 2 shows flow chart diagram. The low AGR group was defined as patients who showed a lower preoperative AGR (< 1.47) and included 1,143 patients (38.5%). The high AGR (≥ 1.47) group was defined as the remaining 1,827 patients (61.5%).

**Figure 3:**
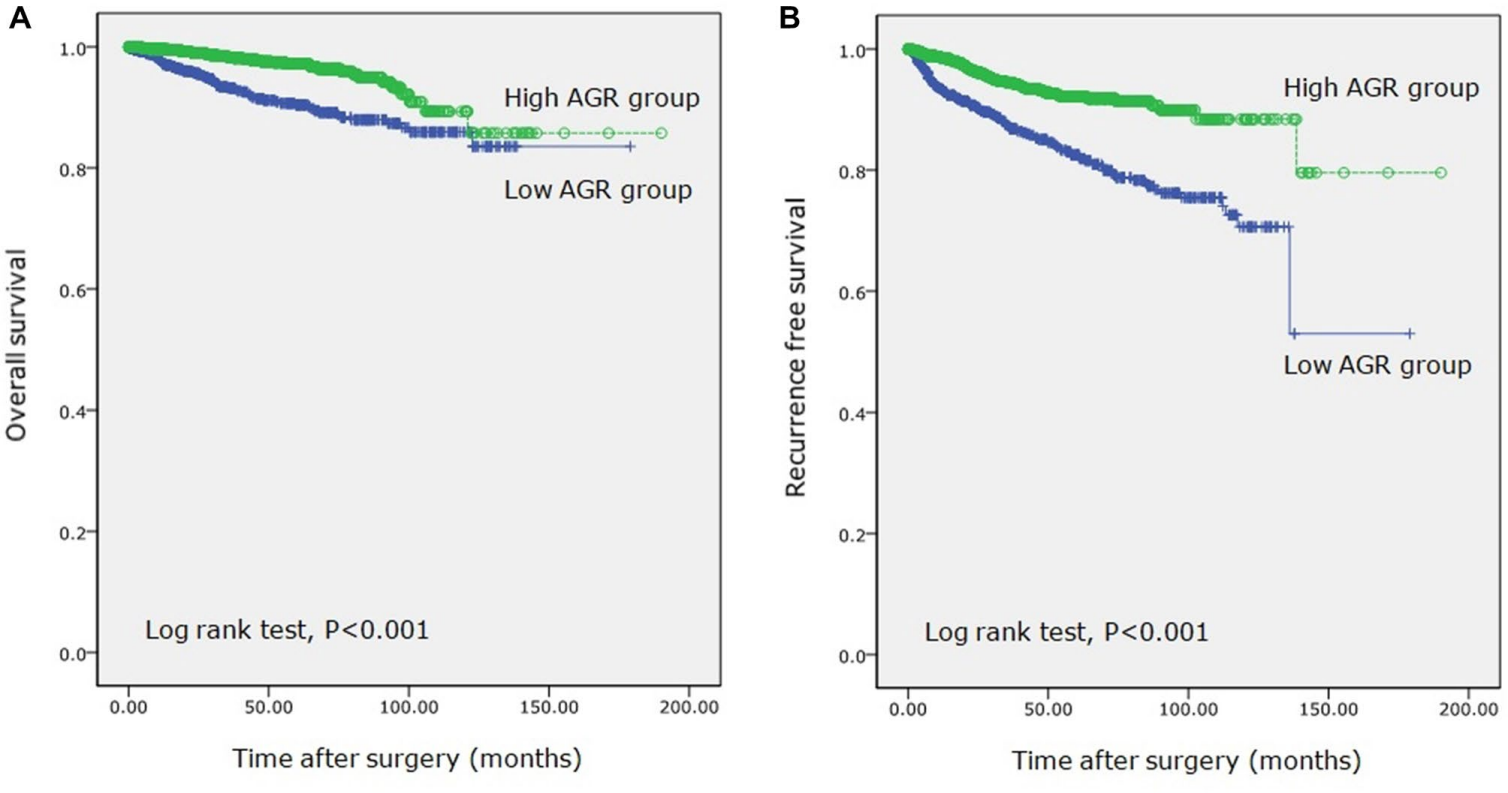
Kaplan–Meier curves of OS (**A**) and RFS (**B**) according to pretreatment AGR. OS, overall survival; RFS, recurrence-free survival; AGR, albumin–globulin ratio.

Kaplan–Meier curves indicated that patients in the low AGR group had a significantly lower rate of OS relative to those in the high AGR group (93.6% vs. 97.9%, respectively; P < 0.001) (Fig. 3A) along with a lower rate of RFS (87.7% vs. 95.3%, respectively; P < 0.001) (Fig. 3B).

Table 1 demonstrates the basic clinical characteristics of patients between the two groups. The mean follow-up period was 26.0 months (range, 9.0–59.0 months). The mean age was 55.6 ± 13.2 years. Patients belonging to the low AGR group were significantly older than patients belonging to the high AGR group (58.7 ± 12.9 years vs. 53.7 ± 13.0 years; P < 0.001). Mean total protein, serum albumin, and AGR were 7.3 ± 0.6 g/L, 4.4 ± 0.4 g/L, and 1.54 ± 0.29, respectively. Total protein in the low AGR group was significantly higher than the high AGR group (7.4 ± 0.6 vs. 7.2 ± 0.5; P < 0.001). Serum albumin (4.1 ± 0.4 vs. 4.5 ± 0.3; P < 0.001) and AGR (1.26 ± 0.18 vs. 1.72 ± 0.20; P < 0.001) in the low AGR group was significantly lower than the high AGR group. The mean body mass index (BMI) was 24.5 ± 3.3 kg/m^2^. The number of patients with a higher American Society of Anesthesiology (ASA) score (≥ 3 points) was significantly greater in the low AGR group than in the high AGR group (6.8% vs. 3.3%; P < 0.001). A total of 30.8% of patients were female. The ratio of females was significantly greater in low AGR group compared with the high AGR group (36.0% vs. 27.6%; P < 0.001). A total of 684 patients (23.0%) presented with symptoms at diagnosis. The low AGR group had a significantly higher rate of symptoms (28.9% vs. 19.4%; P < 0.001). The incidence of hypertension and diabetes mellitus was 39.7% and 15.6%, respectively. Patients belonging to the low AGR group had a significantly higher incidence of hypertension (44.3% vs. 36.9%; P < 0.001) and diabetes mellitus (17.8% vs. 14.2%; P = 0.009) than patients belonging to the high AGR group. There was no significant difference in tumor laterality between the groups. Partial nephrectomy was performed on 480 patients (38.4%). In the high AGR group, partial nephrectomy was carried out on a significantly greater percentage of patients (39.1% vs. 54.1%; P < 0.001). The OS and RFS rates for the total population were 3.7% (n = 111) and 7.6% (n = 227), respectively, while, in the low AGR group, the rates of both OS (6.4% vs. 2.1%; P < 0.001) and RFS (12.3% vs. 4.7%; P < 0.001) were significantly higher than in the high AGR group.

**Table 1:** Clinical characteristics of patients.

Variable		Total (n = 2,970)		Low AGR group (n = 1,143) AGR < 1.47		High AGR group (n = 1,827) AGR ≥ 1.47	P value
Follow up (months) (median [IQR])		26.0 (9.0–59.0)		24.0 (8.0–61.0)		27.0 (10.0–58.3)	0.807
Age (years)		55.6 ± 13.2		58.7 ± 12.9		53.7 ± 13.0	< 0.001
Total protein (g/L)		7.3 ± 0.6		7.4 ± 0.6		7.2 ± 0.5	< 0.001
Serum albumin (g/L)		4.4 ± 0.4		4.1 ± 0.4		4.5 ± 0.3	< 0.001
AGR		1.54 ± 0.29		1.26 ± 0.18		1.72 ± 0.20	< 0.001
Body mass index (kg/m^2^)		24.5 ± 3.3		24.5 ± 3.5		24.6 ± 3.2	0.384
**ASA score**							< 0.001
0–1		1,148 (38.7)		349 (30.5)		799 (43.7)	
2		1,684 (56.7)		716 (62.6)		968 (53.0)	
≥ 3		138 (4.6)		78 (6.8)		60 (3.3)	
**Sex**							< 0.001
Male		2,055 (69.2)		732 (64.0)		1,323 (72.4)	
Female		915 (30.8)		411 (36.0)		504 (27.6)	
**Symptom presentation at the time of diagnosis**							< 0.001
Incidental		2,286 (77.0)		813 (71.1)		1,473 (80.6)	
Symptomatic		684 (23.0)		330 (28.9)		354 (19.4)	
Hypertension		1,180 (39.7)		506 (44.3)		674 (36.9)	< 0.001
Diabetes mellitus		462 (15.6)		203 (17.8)		259 (14.2)	0.009
**Laterality**							0.807
Left		1,429 (48.1)		547 (47.9)		882 (48.3)	
Right		1,541 (51.9)		596 (52.1)		945 (51.7)	
**Type of surgery**							< 0.001
Radical nephrectomy		769 (61.6)		696 (60.9)		73 (45.9)	
Partial nephrectomy		480 (38.4)		447 (39.1)		33 (54.1)	
Overall death		111 (3.7)		73 (6.4)		38 (2.1)	< 0.001
Recurrence		227 (7.6)		141 (12.3)		86 (4.7)	< 0.001

Values are presented as mean ± standard deviation or number (%) unless otherwise indicated.

IQR, interquartile range; AGR: albumin–globulin ratio.

Table 2 demonstrates the pathologic characteristics of patients between the two groups. Tumor location (exophytic, mesophytic, endophytic, or hilar) was not different between two groups. 84 patients (2.8%) presented with sarcomatoid differentiation. The incidence of sarcomatoid differentiation in the low AGR group was significantly higher than that of the high AGR group (5.0% vs. 1.5%, respectively; P < 0.001). Seventeen patients (0.6%) showed a positive surgical margin. Tumor necrosis was found in 495 patients (16.6%). The incidence of tumor necrosis was significantly higher in the low AGR group. Meanwhile, 163 patients (5.5%) showed lymphovascular invasion (LVI) and the incidence of LVI was significantly higher in the low AGR group. Capsular invasion was observed in 587 patients (19.8%). Non-clear RCC accounted was observed in 616 patients (20.7%). The histologic subtype (clear cell RCC vs. non-clear) between the two groups was not statistically different. In the low AGR group, patients with a higher pathologic T-stage and Fuhrman nuclear grade were significantly greater in number than in the high AGR group.

**Table 2:** Pathologic characteristics of patients.

Variable	Total (n = 2,970)	Low AGR group (n = 1,143) AGR < 1.47	High AGR group (n = 1,827) AGR ≥ 1.47	P value
**Tumor location**				0.559
Exophytic	1,661 (56.1)	632 (56.2)	1,029 (56.3)	
Mesophytic	323 (10.9)	126 (11.0)	197 (10.8)	
Endophytic	669 (22.6)	247 (21.6)	422 (23.1)	
Hilar	307 (10.3)	128 (11.2)	179 (9.0)	
**Sarcomatoid differentiation**				< 0.001
No	2,886 (97.2)	1,086 (95.0)	1,800 (98.5)	
Yes	84 (2.8)	57 (5.0)	27 (1.5)	
**Surgical margin**				0.807
Negative	2,953 (99.4)	1,136 (99.4)	1,817 (99.5)	
Positive	17 (0.6)	7 (0.6)	10 (0.5)	
**Necrosis**				< 0.001
No	2,475 (83.3)	909 (79.5)	1,566 (85.7)	
Microscopic	250 (8.4)	76 (6.6)	174 (9.5)	
Macroscopic	245 (8.2)	158 (13.8)	87 (4.8)	
**Lymphovascular invasion**				< 0.001
No	2,807 (94.5)	1,045 (91.4)	1,762 (96.4)	
Yes	163 (5.5)	98 (8.6)	65 (3.6)	
**Capsular invasion**				0.673
No	2,383 (80.2)	913 (79.9)	1,470 (80.5)	
Yes	587 (19.8)	230 (20.1)	357 (19.5)	
**Histology**				0.959
Clear-cell	2,353 (79.3)	905 (79.2)	1,448 (79.3)	
Non-clear cell	616 (20.7)	237 (20.8)	379 (20.7)	
**Pathological T stage**				< 0.001
T1	2,399 (80.8)	829 (72.5)	1,570 (85.9)	
T2	211 (7.1)	110 (9.6)	101 (5.5)	
T3	342 (11.5)	190 (16.6)	152 (8.3)	
T4	18 (0.6)	14 (1.3)	4 (0.3)	
**Fuhrman nuclear grade**				< 0.001
G1	116 (3.9)	32 (2.8)	84 (4.6)	
G2	1,452 (48.9)	509 (44.5)	943 (51.6)	
G3	1,222 (41.1)	491 (43.0)	731 (40.0)	
G4	180 (6.1)	111 (9.7)	69 (3.8)	

Values are presented as number (%).

Table 3 shows univariate and multivariate analysis outcomes for overall death. Per these analyses, age [hazard ratio (HR): 1.028; 95% confidence interval (CI): 1.011–1.045; P = 0.001], sex (HR: 0.476, 95% CI 0.272–0.833; P = 0.009), BMI (HR: 0.934, 95% CI 0.874–0.998; P = 0.044), microscopic tumor necrosis findings (HR: 2.430, 95% CI 1.425–4.145; P = 0.001), LVI (HR: 2.327, 95% CI 1.339–4.046; P = 0.003), pathologic T-stage (HR: 2.036, 95% CI 1.194–3.473; P = 0.009), and AGR (HR: 0.558, 95% CI 0.359–0.867; P = 0.010) were independent predictive components of overall death.

**Table 3:** Univariate and multivariate analysis of overall death.

Variable	Univariate analysis		Multivariate analysis	
	HR (95% CI)	*P* value	HR (95% CI)	*P* value
Age	1.043 (1.026–1.060)	< 0.001	1.028 (1.011–1.045)	0.001
Sex (female vs. male)	0.621 (0.395–0.976)	0.038	0.476 (0.272–0.833)	0.009
Body mass index	0.933 (0.882–0.987)	0.016	0.934 (0.874–0.998)	0.044
Symptom presentation (no vs. yes)	2.265 (1.550–3.285)	< 0.001	1.308 (0.853–2.006)	0.218
Surgical margin (no vs. yes)	3.521 (0.868–14.283)	0.078	1.074 (0.146–7.920)	0.944
**Necrosis**				
No vs. microscopic	3.147 (1.929–5.134)	< 0.001	2.430 (1.425–4.145)	0.001
No vs. macroscopic	3.726 (2.321–5.982)	< 0.001	1.529 (0.867–2.695)	0.142
Lymphovascular invasion (no vs. yes)	6.233 (4.052–9.589)	< 0.001	2.327 (1.339–4.046)	0.003
Capsular invasion (no vs. yes)	1.843 (1.214–2.799)	0.004	0.948 (0.569–1.580)	0.837
T-stage (T1 & T2 vs. T3 & T4)	5.914 (4.064–8.604)	< 0.001	2.036 (1.194–3.473)	0.009
Fuhrman grade (1 & 2 vs. 3 & 4)	2.669 (1.782–4.000)	< 0.001	1.544 (0.980–2.434)	0.061
AGR (low vs. high)	0.352 (0.237–0.522)	< 0.001	0.558 (0.359–0.867)	0.010

Separately, Table 4 presents univariate and multivariate analysis results for recurrence. Symptom presentation at diagnosis (HR: 1.577, 95% CI 1.178–2.110; P = 0.002), microscopic tumor necrosis findings (HR: 1.612, 95% CI 1.074–2.418; P = 0.021), macroscopic tumor necrosis findings (HR: 2.085, 95% CI 1.455–2.988; P < 0.001), LVI (HR: 2.049, 95% CI 1.400–2.499; P < 0.001), pathologic T-stage (HR: 2.357, 95% CI 1.645–3.378; P < 0.001), Fuhrman grade (HR: 2.029, 95% CI 1.477–2.788; P < 0.001), and AGR (HR: 0.526, 95% CI 0.391–0.709; P < 0.001) were independent predictive factors for recurrence.

**Table 4:** Univariate and multivariate analysis of recurrence.

Variable	Univariate analysis		Multivariate analysis	
	HR (95% CI)	*P* value	HR (95% CI)	*P* value
Age	1.014 (1.003–1.025)	0.011	0.997 (0.985–1.008)	0.556
Sex (female vs. male)	0.807 (0.601–1.084)	0.155	0.988 (0.723–1.350)	0.942
Body mass index	0.949 (0.912–0.988)	0.010	0.966 (0.924–1.010)	0.126
Symptom presentation (no vs. yes)	2.720 (2.095–3.532)	< 0.001	1.577 (1.178–2.110)	0.002
Surgical margin (no vs. yes)	1.588 (0.394–6.391)	0.515	1.184 (0.289–4.841)	0.815
**Necrosis**				
No vs. microscopic	2.043 (1.372–3.043)	< 0.001	1.612 (1.074–2.418)	0.021
No vs. macroscopic	5.243 (3.853–7.133)	< 0.001	2.085 (1.455–2.988)	< 0.001
Lymphovascular invasion (no vs. yes)	6.476 (4.755–8.819)	< 0.001	2.049 (1.400–2.499)	< 0.001
Capsular invasion (no vs. yes)	2.298 (1.737–3.040)	< 0.001	1.206 (0.868–1.674)	0.264
T-stage (T1 & T2 vs. T3 & T4)	6.339 (4.871–8.249)	< 0.001	2.357 (1.645–3.378)	< 0.001
Fuhrman grade (1 & 2 vs. 3 & 4)	3.384 (2.541–4.508)	< 0.001	2.029 (1.477–2.788)	< 0.001
AGR (low vs. high)	0.374 (0.286–0.490)	< 0.001	0.526 (0.391–0.709)	< 0.001

## Discussion

In this relatively big cohort study with retrospective nature, we revealed that preoperative AGR can be a useful predictive factors for estimating OS and RFS in patients with RCC. Although a few researchers have studied the association between AGR and prognosis of RCC patients, to the best of our knowledge, few studies have been carried out in a large cohort with a multicenter design to assess the pretreatment AGR as a predictive component for estimating OS and RFS in RCC patients.

Recent advances in cancer biology has revealed that a systemic malnutrition and inflammation are associated with poor prognosis^21^. Up to now, numerous proteins (more than 10,000) have been found in human plasma^22,23^; of which albumin comprises more than half^11^. Serum albumin is a water-soluble hepatic protein and a transporting molecule for several hormones, minerals, and fatty acids while also helping to maintain the oncotic pressure of the capillaries^24^. Its half-life is roughly 2 to 3 weeks. Further, albumin plays an antioxidative role in plasma and the interstitial space and provides amino acids for matrix deposition and cell proliferation^15,25^. In an inflammatory state, hypoalbuminemia is induced in response to the increased capillary escape of serum albumin into the interstitium^15^. Serum albumin can be classified as a negative acute-phase protein and is regulated by various inflammatory conditions^26^. It is understood that increased concentrations of acute-phase proteins and immunoglobulins can increase the serum globulin concentration; these responses correlate with an inflammatory state. A few studies have indicated that serum globulin presents an association with prognosis in patients with cancer^27,28^. Globulin contains inflammatory mediators such as chemokines, cytokines, and other small inflammatory proteins^22,23,29^. The local or systemic immune response in cancer-related inflammation are associated with an increased production of these inflammatory mediators^29,30^. Recently, the relationship between pretreatment AGR and various malignancy has attracted the attention of many scientists. The AGR is mostly adopted as a medical measurement approach in the context of numerous immune-proliferative diseases^31^. However, albumin and globulin have been separately associated with a broad spectrum of long-term illnesses. In particular, increase of serum globulins can be associated with diabetes mellitus, rheumatoid disease, chronic liver disease, nephrotic syndrome, and cancer; whereas decrease of albumin can be associated with chronic liver disease, chronic infections, and nephrotic syndrome^32,33^.

Recent study has exhibited that a low AGR has association with rate of tumor incidence and mortality in health check-up population^34^. In 2014, Suh et al^34^. performed the retrospective cohort trial on 26,974 healthy people over the age of 30. They demonstrated that a low AGR was related with the occurrence and death of cancer, in the short term as well as long-term, in a generally healthy check-up population. Low AGR could represent a general pathway for carcinogenesis because chronic inflammation is associated with carcinogenesis based on observational results of cancer developing from inflammatory cells which is existing in tumor sites^30,35,36^. Furthermore, some researches have showed that low AGR has association with worse prognosis in breast^16^, colorectal^37^, nasopharyngeal^38^, and lung cancers^39^. Therefore, AGR could serve as a marker of the cancer-related inflammatory response^15,40,41^.

In other word, a low AGR can be a risk factor for increasing malignancy occurrence and death in healthy populations^34^. In a meta-analysis on human cancer, a low AGR has association with poor OS, disease-free survival (DFS), and increased 5-year mortality^18^. The prognostic value of low AGR in RCC patients is thought to be correlated with the potential mechanisms relating to inflammation and nutrition in the cancer environment. Despite that proper nutrition before and after surgery is important for cancer patients, in the clinic, there is still a relatively large number of cancer patients who visit while showing malnutrition. Furthermore, malnutrition often causes cancer cachexia to develop and it is associated with cancer recurrence and progression. Chronic inflammation is present in almost all cancer environments^42^ as malignant tumor cells release many inflammatory factors during angiogenesis, tissue remodeling, and rehabilitation^30^. As such, changes in inflammatory factors including vascular endothelial growth factor; tumor necrosis factor; and interleukin-1, -6, -8, in the tumor microenvironment facilitate tumor growth and distant metastasis^36,42^. As such, poor nutritional status is strongly correlated with the recurrence and progression of cancer.

To date, three single-center-based researches have evaluated the low preoperative AGR as a poor predictive factor in RCC patients^17,19,20^. Chen et al.^17^ carried out a retrospective cohort study that included 416 localized and locally advanced clear-cell RCC patients. In their research, the best cutoff value of AGR was 1.22, which is lower than that used in the present study. These authors suggested that low AGR is an independent predictive component for estimating OS (HR: 6.53, 95% CI 3.04–14.04; P < 0.001) as well as cancer-specific survival (HR: 8.81, 95% CI 3.89–19.93; P < 0.001). Elsewhere, He et al.^19^ conducted a relatively large retrospective cohort study involving 895 RCC patients of all cancer stages, where the best cutoff value of AGR was calculated to be 1.47, which is the same as that used in the present study. In this study, a low pretreatment AGR had an association with old age, lower albumin, low hemoglobin, high total protein, low BMI, and advanced disease stage. In accordance with the multivariate Cox regression analysis, a low pretreatment AGR also showed an association with increased mortality and was an independent predictive component for estimating poor OS (HR: 0.63, 95% CI 0.43–0.93; P = 0.022). Koparal et al.^20^ reported, in a retrospective cross-sectional study of 162 clear-cell RCC cases, that the best cutoff value of AGR was 1.40. Further, the DFS and OS of the low AGR group were significantly lower in accordance with the Kaplan–Meier analysis (all P < 0.05). However, considering that the numbers of cases in the previous two studies by Chen et al. and Koparal et al., respectively, were relatively small, it is thought to be hasty to conclude that there is any association between low AGR and RCC prognosis. Furthermore, while He et al.’s study included a relatively large number of patients, it was a single-center investigation and did not analyze pathological outcomes such as tumor necrosis, LVI, capsular invasion, and positive surgical margin, which are strongly associated with the prognosis of patients with RCC. Unlike the three studies described above, our study included a large number of patients from multiple centers and analyzed various clinical and pathologic characteristics. This constitutes the biggest advantage of our research.

In present study, the best cut-off value of AGR was 1.47 in accordance with the ROC curve. A low preoperative AGR had a significant statistical correlation with the advanced pathologic stage and Fuhrman nuclear grade, tumor necrosis, LVI, sarcomatoid differentiation, and the presence of symptoms at diagnosis (P < 0.001). The analysis of Kaplan–Meier survival revealed that OS and RFS were lower in the low AGR group patients (all P < 0.001). In accordance with the multivariate Cox regression analysis, the high AGR group exhibited a decreased risk of mortality and decreased risk of recurrence, while low AGR was an independent predictive value for estimating both overall death (HR: 0.558, 95% CI 0.359–0.867; P = 0.010) and recurrence (HR: 0.526, 95% CI 0.391–0.709; P < 0.001).

The limitations of the present study include the retrospective data collection and comparatively short follow-up period (26 month). Retrospective design may introduce sampling bias. For example, age, sex, and BMI were independent factors for predicting poor OS but not RFS. Similarly, symptom presentation at diagnosis and Fuhrman nuclear grade showed significant associations with RFS but not OS. Due to the retrospective nature of this study, selection bias was inevitable and this study involved only a short term follow-up period, so its conclusions must be carefully judged. Furthermore, there was no information about blood test variables, containing the platelet count and neutrophil–lymphocyte ratio, which can affect OS and RFS. To overcome these weak points, researchers from multiple institutions included in the present study will monitor the study population continuously. Finally, a multi-institutional database of the present study did not contain any information of patients accompanied by severe illness or elderly patients with high comorbidities who could not be treated with surgery. This could lead to incomplete observations of the whole RCC spectrum in Korea.

In spite of the limitations of our research described above, this study may have many clinical implications. First of all, to our knowledge, this is the first multi-institutional study with a large cohort to demonstrate that low AGR is an independent prognostic factor for predicting poor OS and RFS in patients with RCC. Also, preoperative serum albumin and globulin can be measured easily and inexpensively. Due to these advantages, AGR has some potential as a convenient and simple marker for urologists to adopt in counseling RCC patients during clinical decision-making. Finally, the preoperative evaluation of nutritional status and supplementation to achieve adequate nutrition should be conducted in patients with low AGR (< 1.47). In the near future, further large-scale population-based prospective multi-institutional studies involving factors that may influence the outcomes of kidney cancer should be performed.

## Conclusions

In conclusion, AGR is an important prognostic determinant in non-metastatic RCC. A low AGR (< 1.47) was related with older age, higher ASA score, the female sex, symptom presentation at the time of diagnosis, and preexisting comorbidities such as hypertension and diabetes mellitus. Sarcomatoid differentiation, tumor necrosis, LVI, pathologic T stage and Fuhrman nuclear grade were also significantly correlated with a low AGR. Furthermore, the low value of AGR was a significant predictive factors of OS and RFS. Preoperative AGR is an easy-to-use and inexpensive method that can be used to estimate prognosis in RCC patients treated with surgical management. Therefore, these findings may help urologists to give preoperative advice to patients with a low AGR before surgical management.

## Materials and methods

### Study design

The database contained clinical characteristics of patients, including follow-up months, age at diagnosis, concentrations of serum total protein and albumin, AGR, sex, BMI, ASA score, presence of symptoms at diagnosis, comorbidities (diabetes and hypertension), surgical method (radical nephrectomy or partial nephrectomy), recurrence rate, and overall death rate. The pathologic characteristics of patients were as follows: tumor location, presence of sarcomatoid differentiation, surgical margin status, tumor necrosis, LVI, capsular invasion, pathologic tumor stage, Fuhrman nuclear grade, and histologic subtype^43,44^. Especially, the classification of tumor location was based on our previous research using the Korean RCC (KORCC) database^45,46^. In particular, hilar RCC was defined as a tumor located just adjacent to the main renal vessel or its segmental branches without invasion^47^. The seventh edition of the American Joint Committee on Cancer classification system was used for evaluating pathological staging^43,48^, while the Fuhrman grading system was chosen to evaluate nuclear differentiation^49,50^. To protect the confidentiality of patients, personal information, such as resident registration numbers and hospital identification numbers, was eliminated^43,44^. The requirements for enrollment were complete information about preoperative serum total protein and albumin and a diagnosis of clinical and pathological non-metastatic RCC. All patients were assessed using routine laboratory tests. In addition, imaging studies, including chest X-rays and abdominal computed tomography (CT), were performed. Certain data were excluded from the analysis due to insufficient or missing variables (AGR data missing, n = 3,706). Cases of bilateral renal tumor, solitary kidney, or recurrent disease (n = 352) were excluded. Consequently, the final analysis included 2,970 consecutive patients with non-metastatic RCC who received surgical management (Fig. 2). Imaging investigation (abdomen and chest CT and bone scan) was used to identify recurrence. Recurrence was defined as local recurrence, metastasis to the lymph node, or distant metastasis.

### Statistical analyses

ROC curves were developed to ascertain the pretreatment AGR cutoff value. Clinical, surgical, and pathological parameters between the two groups were compared using the Student’s t-test (continuous variables) and the chi-squared or Fisher’s exact test (categorical variables) where appropriate. RFS and OS were evaluated using the Kaplan–Meier method with the log-rank test. Univariate and multivariate Cox regression analyses were conducted to discern the predictive factors that affect RFS and OS and to generate the HRs with 95% CIs. Statistical analysis was performed using the Statistical Package for the Social Sciences version 16.0 for Windows (IBM Corp., Armonk, NY, USA) and P*-*values of less than 0.05 were considered to be statistically significant.

### Ethical approval

From the related institutional review boards of each participating center, approval for the study was achieved. Data from 7,136 patients who underwent partial or radical nephrectomy for a kidney mass from March 1999 to December 2015 from the Korean RCC database at eight academic centers were analyzed. After the data were collected retrospectively and pooled centrally, the institutional review board (IRB) of Kyungpook National University, School of Medicine, Daegu, Republic of Korea (IRB no. KNUH 2016-05-021) approved the present trial. The study was conducted in agreement with the relevant laws and regulations, good clinical practices, and ethical principles as described in the Declaration of Helsinki. The need for informed consent was waived by the aforementioned IRB due to the retrospective nature of the study.

## Data Availability

The data supporting the findings of this study are available from the KORCC group but restrictions apply to the availability of these data, which were used under license for the current study and so are not publicly available. Data are, however, available from the authors upon reasonable request and with permission from the KORCC group.
